# Examining COVID-19 vaccine uptake and attitudes among 2SLGBTQ+ youth experiencing homelessness

**DOI:** 10.1186/s12889-022-12537-x

**Published:** 2022-01-18

**Authors:** Alex Abramovich, Nelson Pang, Sharumathy Kunasekaran, Amanda Moss, Tara Kiran, Andrew D. Pinto

**Affiliations:** 1 Institute for Mental Health Policy Research, Centre for Addiction and Mental Health, 33 Ursula Franklin Street, Toronto, ON M5S 2S1 Canada; 2 Dalla Lana School of Public Health, University of Toronto, Toronto, Ontario Canada; 3 Department of Psychiatry, University of Toronto, Toronto, Ontario Canada; 4 Department of Family and Community Medicine, St. Michael’s Hospital, Toronto, Ontario Canada; 5 Department of Family and Community Medicine, University of Toronto, Toronto, Ontario Canada; 6 MAP Centre for Urban Health Solutions, St. Michael’s Hospital, Toronto, Ontario Canada; 7 Institute of Health Policy, Management, and Evaluation, University of Toronto, Toronto, Ontario Canada

**Keywords:** 2SLGBTQ+ youth, Gender identity, Sexual orientation, Homelessness, COVID-19 vaccines, Mixed-methods

## Abstract

**Objectives:**

The COVID-19 pandemic has disproportionately impacted 2SLGBTQ+ youth experiencing homelessness. Little is known about vaccine attitudes and uptake among this population. To address this, the objectives of this study were to explore this group’s COVID-19 vaccine attitudes, and facilitators and barriers impacting vaccine uptake.

**Methods:**

2SLGBTQ+ youth experiencing homelessness in the Greater Toronto Area were recruited to participate in online surveys assessing demographic characteristics, mental health, health service use, and COVID-19 vaccine attitudes. Descriptive statistics and statistical tests were used to analyze survey data to explore variables associated with vaccine confidence. Additionally, a select group of youth and frontline workers from youth serving organizations were invited to participate in online one-on-one interviews. An iterative thematic content approach was used to analyze interview data. Quantitative and qualitative data were merged for interpretation by use of a convergent parallel analytical design.

**Results:**

Ninety-two youth completed surveys and 32 youth and 15 key informants participated in one-on-one interviews. Quantitative and qualitative data showed that the majority of 2SLGBTQ+ youth experiencing homelessness were confident in the COVID-19 vaccine; however, numerous youth were non-vaccine confident due to mistrust in the healthcare system, lack of targeted vaccine-related public health information, concerns about safety and side effects, and accessibility issues. Solutions to increase vaccine confidence were provided, including fostering trust, targeted public health messaging, and addressing accessibility needs.

**Conclusion:**

Our study highlights the need for the vaccine strategy and rollouts to prioritize 2SLGBTQ+ youth experiencing homelessness and to address the pervasive health disparities that have been exacerbated by the pandemic.

**Supplementary Information:**

The online version contains supplementary material available at 10.1186/s12889-022-12537-x.

## Background

The COVID-19 pandemic has disproportionately impacted marginalized populations, including 2-spirit, lesbian, gay, bisexual, transgender, queer, and questioning (2SLGBTQ+) youth, particularly 2SLGBTQ+ youth experiencing homelessness, who make up 20-40% of the homeless youth population across North America [1–3]. Recent research has suggested that 2SLGBTQ+ individuals have been more likely to report COVID-19 related mental health impacts, including depression, suicide, and substance use, compared to cisgender and heterosexual individuals, and these risks are greatly increased among those who experience added layers of marginalization, including homelessness [4, 5]. This is largely due to experiences of stigma, discrimination, violence, familial rejection, and the closure of social support services and delay of gender affirming medical care [4, 5]. Loss of access to inclusive and affirming medical and social supports are cause for concern, as they have been found to be key protective factors for suicide, self-harm, and depression among 2SLGBTQ+ individuals [4].

Vaccinations have become a vital mechanism to curb the spread of COVID-19 [6]; however, previous research suggests that some groups are less likely than others to receive immunization in general, including youth and individuals experiencing homelessness [7]. This is particularly concerning given that people experiencing homelessness often have poorer health and are more susceptible to infectious diseases [6, 7]. Although this group faces unique health concerns, there are many barriers to immunization, including missing identification, lack of permanent address, stigma, and discrimination [7]. Structural factors, such as health inequalities, socioeconomic disadvantages, and systemic racism may also impact vaccine confidence [8]. Additionally, immunization may be viewed as low priority for youth experiencing homelessness, who are often in “survival mode”, and consistently focused on getting their basic needs met [9]. Little is known about vaccine attitudes and uptake among 2SLGBTQ+ youth experiencing homelessness. The objective of this study was to examine the impact of COVID-19 among 2SLGBTQ+ youth and young adults at risk of, and experiencing, homelessness, and to explore their attitudes towards COVID-19 vaccines, vaccine uptake, and intentions to get vaccinated.

## Methods

This mixed-methods study is part of a larger longitudinal study utilizing a convergent parallel design, which involves the simultaneous collection of quantitative and qualitative data, [10] to understand the impacts of the COVID-19 pandemic on 2SLGBTQ+ youth and young adults at risk of, and experiencing, homelessness in the Greater Toronto Area (GTA) and surrounding areas. A Community Advisory Board (CAB), made up of frontline staff and management from youth serving organizations, advised on numerous aspects of the project including: study design, interview guide, survey development, analysis and knowledge translation. Study procedures were approved by the Centre for Addiction and Mental Health (CAMH) Research Ethics Board (REB #102/2020).

### Context and setting

Data collection occurred between January 2021 to June 2021, during the second and third waves of COVID-19 in Ontario, Canada, at a time when the province was moving in and out of lockdown restrictions and stay-at-home orders. Ontario’s three step plan for the COVID-19 vaccination rollout also took place during this time [11].

In January 2021 (when data collection began), Ontario was in the midst of the second wave of the COVID-19 pandemic (declared in September 2020). At this time, Ontario was also in a strict provincewide lockdown that began in December 2020. Simultaneously, the province was in Phase 1 of the vaccine rollout, which began in December 2020, and prioritized health care workers, congregate living for seniors, First Nations, Métis and Inuit populations, adult chronic home care recipients, and adults aged 80+. Youth over the age of 18 were eligible to receive a `vaccine in Phase 1 if they identified with one of the listed categories (e.g., health care worker, Indigenous, etc.); however, vaccines for individuals under the age of 18 were not yet approved in Canada (i.e., youth who were under 18 years old and identified with one of the listed categories were not eligible for a vaccine). In February 2021, Ontario was recovering from the second wave of the COVID-19 pandemic and COVID-19 cases were steadily decreasing. The stay-at-home order was partially lifted in select regions of the province. Ontario remained in Phase 1 of vaccine rollout during this time. In the beginning of March 2021, remaining regions of Ontario exited out of stay-at-home orders; however, COVID-19 cases continued to rise throughout March and a third wave of the COVID-19 pandemic was declared. Ontario remained in Phase 1 of the vaccine rollout at this time. In April 2021, Ontario reached its peak number of COVID-19 cases and a second province-wide lockdown and stay-at-home order was put into effect. At this time, Ontario moved into Phase 2 of the vaccine rollout, which included adults aged 55+, individuals with certain health conditions, those who cannot work from home, people who live in hot spot regions and high-risk congregate settings (such as shelters and group homes). Youth over the age of 18 were eligible for a vaccine in Phase 2 if they identified with one of the listed categories (e.g., cannot work from home, live in a congregate setting, etc.); however, vaccines for individuals under the age of 18 were not yet approved in Canada. In May 2021, Ontario was recovering from the third wave of the virus and COVID-19 cases were steadily decreasing. Ontario remained in Phase 2 of vaccine rollout at this time. At the end of May 2021, the vaccine was approved for individuals aged 12 and over (i.e., youth aged 12 and over became eligible for a vaccine in Phase 2 if they identified with one of the aforementioned listed categories [e.g., cannot work from home, live in a congregate setting, etc.]). In June 2021 (when data collection ceased) COVID-19 cases continued to plummet and Ontario entered into Step 1, and shortly after - Step 2, of the Roadmap to Reopen plan (a 3-step plan to reopen the province). Ontario remained in phase 2 of the vaccine rollout at this time.

### Participants

Ninety-two 2SLGBTQ+ youth and young adults at risk of, and experiencing, homelessness were recruited to participate in this study. This study includes young people up to the age of 29, in line with the Government of Canada’s definition of youth (up to the age of 29) [1]. Participant enrollment occurred on an ongoing basis, beginning in January 2021 and concluding in June 2021. Criteria for inclusion were: self-identify as 2SLGBTQ+; aged 14-29; at risk of, or experiencing, homelessness (defined as individuals living with unsupportive family or in housing situations lacking security and/or stability; struggling to pay rent; staying at a shelter or housing program; living independently of parents/caregivers, but unable to secure stable, safe, or consistent housing); living in the GTA or surrounding areas (Toronto, Durham, Peel, York Region, Hamilton, Barrie, Guelph, Waterloo).

The research team used their extensive network of community and policy partnerships to recruit 15 key informants from ten youth serving organizations across Ontario to participate in semi-structured, in-depth, online, one-on-one interviews. Key informants were frontline staff and management at youth serving organizations, housing and shelter services, child protection agencies, and government shelter operations.

## Quantitative methods

### Recruitment and sampling

We recruited study participants by using a convenience sampling method and collaborating with youth serving organizations to refer eligible participants from their programs to the research study. The research team requested to host information sessions online at the youth serving organizations in order to share study details directly with potential participants and answer any questions. The information sessions were organized and executed in collaboration with the staff from the youth serving organizations (e.g., the staff invited their program participants to the session, etc.) and was led by a 2SLGBTQ+ Peer Support Worker hired on the research team specifically for participant recruitment efforts. The peer support worker also assisted with the development and circulation of paid social media advertisements on Instagram and Facebook. Individuals interested in participating in the study were asked to contact the research team via email to determine eligibility. The surveys were pilot tested within the research team, with members of the Community Advisory Board, and with several other key stakeholders prior to going live for participants. Participants were compensated with a $35 electronic gift card upon completion of the baseline survey. Findings based on data collected from January 2021 to June 2021 are included in this article.

### Data collection

Interested participants completed a screening survey to determine eligibility and those who met the inclusion criteria were provided with unique survey links. Ninety-two youth completed written informed consent prior to completing the first (baseline) of three online surveys. This article focuses on the first survey from a set of longitudinal surveys over six months.

Information collected in the survey included questions on demographic characteristics, impacts of the COVID-19 pandemic on mental health and health service use, and vaccine attitudes and uptake. Health outcomes were also assessed using validated and standardized measures, including the General Anxiety Disorder-7 item scale (GAD-7) for anxiety, [12, 13] the Patient Health Questionnaire (PHQ-9) for depression, [13, 14] and the CAGE-AID Questionnaire adapted to screen for alcohol and drug use [15, 16]. For the GAD-7, cut-off scores of 5, 10, and 15 represented mild, moderate, and severe anxiety, respectively [12]. For the PHQ-9, cut-off scores of 5, 10, 15, and 20 represented mild, moderate, moderately severe, and severe depression [14]. For the CAGE-AID screening tool, a score of two or more indicated problematic alcohol and/or substance use [15]. Suicidality was measured using a scale derived from a four-item scale previously used in studies with youth [17, 18]. Vaccine confidence was measured utilizing an adapted version of the Vaccination Attitudes Examination Scale to be specific for the COVID-19 vaccine [19]. Lack of confidence in the COVID-19 vaccine was determined if youth were not planning to or were unsure about receiving the COVID-19 vaccine. The survey took approximately 30 min to complete (see Additional file 1 for key measures included in the survey). Survey data was collected and managed using REDCap electronic data software hosted at the Centre for Addiction and Mental Health (CAMH) [20, 21].

### Analysis

Participant responses were carefully reviewed and static factors, such as age and race, were compared between screener, baseline and follow up surveys to determine the legitimacy of participants. Eight participants were removed from the analysis due to major inconsistencies between static factors in survey responses. We included all partially completed questionnaires in the data analysis. All analyses were conducted in R version 3.6.3 [22]. Data was analyzed using descriptive statistics and statistical tests for difference of proportions. Additionally, simple logistic regressions were applied to study the association between the independent variables and COVID-19 vaccine confidence. Quantitative results are reported according to the Checklist for Reporting Results of Internet E-Surveys (CHERRIES) guidelines [23].

## Qualitative methods

### Recruitment and sampling

Upon completion of the baseline survey, all of the individuals who expressed interest in participating in an online one-on-one interview were invited to participate via email and accepted on a first-come basis. Key informants were also invited to participate in an online one-on-one interview via email. Youth interview participants were compensated with a $40 electronic gift card; no compensation was provided to key informants. The youth and key informant interview guides were pilot tested with key stakeholders and within the research team prior to conducting the interviews.

### Data collection

One Research Analyst, with a Master of Social Work degree, conducted in-depth, semi-structured one-on-one interviews (approximately 60 min) online with 32 youth and 15 key informants using a secure video conferencing platform. There was no relationship established between the interviewer and participants prior to the interview. Additional written informed consent was obtained from each participant prior to the beginning of their interview. The interview guide for youth and key informants included questions focused on COVID-19 related challenges, mental health, health and community service use, attitudes towards the COVID-19 vaccine, and vaccine uptake. Interviews were audio-recorded and transcribed verbatim. Field notes were written after the interviews took place. Once it was determined by the research team that qualitative data collection met saturation, data collection was ceased.

### Analysis

Three research team members coded and analyzed the qualitative data, in conjunction with data collection, by use of an iterative thematic content approach [24, 25]. This approach involved open coding to produce numerous key themes that arose from the data. These initial codes were then categorized into major themes and sub-themes. Personal identifiers were removed from the interview transcripts and replaced with pseudonyms to ensure anonymity of participants. All of the names in the results section reflect pseudonyms that were selected by participants. Qualitative results are reported according to the Consolidated Criteria for Reporting Qualitative Studies (COREQ) guidelines [26].

### Mixed-methods data integration

Qualitative and quantitative data were analyzed independently and then combined for interpretation by use of a convergent parallel analytical design [27, 28]. The data sources and methodologies were triangulated to confirm, cross-validate, and corroborate findings within and between participants [24]. The interpreted results of the combined quantitative and qualitative data are presented below.

## Results

Table 1 reports the sociodemographic characteristics of the youth survey participants. Frequencies of under five are not reported to preserve the privacy of participants. Youth had an average age of 20 years, median age of 19; the majority were White 61% (*n* = 56); identified their gender identity as transgender or gender diverse (*n* = 53; ~ 58%); and their sexual orientation as bisexual or pansexual (*n* = 40; ~ 43%).

**Table 1:** Sociodemographic Characteristics of Youth Participants (*n* = 92)

Age Category	n (%)
15-19	51 (55.43%)
20-24	25 (27.17%)
25+	16 (17.39%)
**Gender Identity**	
Cisgender woman	26 (28.26%)
Cisgender man	13 (14.13%)
Transgender woman	12 (13.04%)
Transgender man	14 (15.22%)
Gender diverse (including two-spirit, non-binary, genderfluid)	27 (29.35%)
**Sexual Orientation**	
Asexual	< 5
Bisexual	29 (32.58%)
Demisexual	< 5
Gay	16 (17.98%)
Lesbian	14 (15.73%)
Pansexual	11 (12.36%)
Queer (including fluid)	< 5
Straight/Heterosexual	< 5
Not listed	< 5
**Education**	
Less than high school	38 (41.76%)
Completed high school	35 (38.46%)
Post-secondary	18 (19.78%)
**Ethno-Racial Background** (Select all that apply)	
Asian (East, South, West)	12
Black/African/Caribbean	15
Indigenous	< 5
Latinx	< 5
Mixed-Background	12
White/European	56
**Housing** (Select all that apply)	
A place you rent	22
Parents’/caregivers’ place	29
Friends’ or partners’ place	53
Emergency/domestic violence shelter	17
Supervised residence/transitional housing	34
Public space (vehicle, makeshift shelter, vacant building)	34
Motel or hotel	< 5

Categories reported as < 5 to preserve privacy and confidentiality

Table 2 reports sociodemographic characteristics of youth interview participants. Youth had an average age of 21, median age of 21; the majority were White (*n* = 18; ~ 56%); identified their gender identity as transgender or gender diverse (*n* = 20; ~ 62%); and their sexual orientation as bisexual or pansexual (*n* = 14; ~ 45%). Overall, the sample of interview participants were representative of the larger study population that completed the survey.

**Table 2:** Sociodemographic Characteristics of Interview Participants (*n* = 32)

Age Category	n (%)
15-19	10 (31.25%)
20-24	15 (46.88%)
25+	7 (21.88%)
**Gender Identity**	
Cisgender woman	7 (21.88%)
Cisgender man	< 5
Transgender woman	< 5
Transgender man	7 (21.88%)
Gender diverse (including two-spirit, non-binary, genderfluid)	9 (28.13%)
Not Listed	< 5
**Sexual Orientation**	
Asexual	< 5
Bisexual	10 (31.25%)
Demisexual	< 5
Gay	7 (21.88%)
Lesbian	< 5
Pansexual	< 5
Queer (including fluid)	5 (15.63%)
Straight/Heterosexual	< 5
Not listed	< 5
**Education**	
Less than high school	7 (21.88%)
Completed high school	15 (46.88%)
Post-secondary	10 (31.25%)
**Ethno-Racial Background (Select all that apply)**	
Asian (East, South, West)	6
Black/African/Caribbean	9
Indigenous	< 5
Latinx	< 5
Mixed-Background	12
White/European	18
**Housing (Select all that apply)**	
A place you rent	13
Parents’/caregivers’ place	7
Friends’ or partners’ place	16
Emergency/domestic violence shelter	6
Supervised residence/transitional housing	13
Public space (vehicle, makeshift shelter, vacant building)	9
Motel or hotel	< 5

Categories reported as < 5 to preserve privacy and confidentiality

The majority of participants reported experiencing mental health difficulties throughout the COVID-19 pandemic (See Table 3). A high proportion of youth (*n* = 71; ~ 78%) scored in the severe anxiety range on the General Anxiety Disorder-7 item scale (GAD-7), [12, 13] whereas ~ 59% (*n* = 53) of youth scored in the moderately severe or severe category for depression on the PHQ-9 scale [14]. Approximately 79% (*n* = 72) of youth had engaged in non-suicidal self-injury since the beginning of the pandemic (since March 2020).

**Table 3:** Mental Health of Youth Participants (n = 92)

Depression (past 2 weeks)	n (%)
Minimal	5 (6.56%)
Mild/Moderate	32 (35.56%)
Moderately Severe/Severe	53 (58.88%)
**Anxiety**	
Mild/Moderate	20 (21.98%)
Severe	71 (78.02%)
**Alcohol and substance use (past 2 weeks)**	
Non-problematic	38 (43.68%)
Problematic	49 (56.32%)
**Attempted suicide since COVID-19 (since March 2020)**	
No	60 (65.22%)
Yes	32 (34.78%)
**Non-suicidal self-injury (since March 2020)**	
No	19 (20.88%)
Yes	72 (79.12%)

### COVID-19 vaccine attitudes

Table 4 shares results based on youths’ COVID-19 vaccine attitudes and vaccine uptake. The vast majority (*n* = 68, ~ 75%) of youth reported that they believe that the COVID-19 vaccine can curb the spread of the virus; that they (*n* = 53, ~ 58%) can rely on the vaccine to protect them from the virus; and that they (*n* = 61, ~ 67%) believe that the information they receive about the COVID-19 vaccine is reliable and trustworthy.

**Table 4:** COVID-19 Vaccine Attitudes (*n* = 91)

Do you plan on receiving the COVID-19 vaccine?	n (%)
Yes or have already received it	58 (63.73%)
No	14 (15.38%)
Unsure	19 (20.88%)
**I trust that the information I receive about the COVID-19 vaccine is reliable and trustworthy**	
Strongly disagree/disagree	14 (15.38%)
Neutral	16 (17.58%)
Strongly agree/agree	61 (67.03%)
**I worry about the unknown effects of the COVID-19 vaccine**	
Strongly disagree/disagree	30 (32.97%)
Neutral	16 (17.58%)
Strongly agree/agree	45 (49.45%)
**I can rely on the COVID-19 vaccine to protect me from COVID-19**	
Strongly disagree/disagree	21 (23.08%)
Neutral	17 (18.68%)
Strongly agree/agree	53 (58.24%)
**I believe the COVID-19 vaccine can curb the spread of COVID-19**	
Strongly disagree/disagree	8 (8.79%)
Neutral	15 (16.48%)
Strongly agree/agree	68 (74.72%)

Over-half (n = 53, ~ 58%) of youth reported that they feel safe about receiving the vaccine and ~ 64% (*n* = 58) of youth were planning to or had already been vaccinated, whereas ~ 36% (*n* = 33) of youth were either unsure or not planning to get vaccinated. Participants also expressed concern about the vaccine. For example, ~ 49% (*n* = 45) of youth reported that they feel worried about the unknown effects of the vaccine, as one participant stated:

“*I don't have an argument against the vaccine, it's not like I'm anti-vaccine. I have gotten other vaccines. There's a paranoia within me to not get it. I think it is definitely influenced by media somewhat, like with conversations around side-effects and stuff like that and just with the change in information.”* (Rora, 28 years old)

Some youth also shared uncertainty regarding the legitimacy of the vaccine: “*I find it irrelevant for me to take it. I don’t know because I’ll probably take it if it’s official, like officially a vaccine, like the hepatitis ones in high school and in all the schools, like if it’s certified, I’d take it when it’s actually certified.”* (Anonymous, 17 years old).

It was also noted that trauma and ongoing mental health issues may have impacted participants’ perceptions of COVID-19 and interest in getting vaccinated, such that the risk of infection may not have been as great of a concern for individuals facing multiple intersections of systemic and societal marginalization. For example, one key informant (emergency shelter) stated:

*“I think youth feel that COVID is something that is manageable, in terms of their day-to-day lives...Experiences of violence – whether that’s victimization, whether that’s physical violence, sexual violence, trafficking, experiences of incarceration, you know the trauma associated with homelessness. What I have seen and heard from the youth I support, is that those experiences are more impactful and have more long-term detrimental effects, than their fear of COVID.”*

Several youth also discussed the importance of incentives in motivating people to receive the COVID-19 vaccine: *“Free money, I mean yeah I think that’s a good incentive. If people are going to spread nonsense about how it makes your arm magnetic, maybe paying people to get it will help in that regard.”* (Jasper, 18 years old).

### COVID-19 vaccine confidence

Approximately 36% (*n* = 33) of the 91 youth who completed the vaccine-related survey questions reported that they were unsure or not planning to receive the COVID-19 vaccine, while ~ 64% (*n* = 58) of youth were vaccine confident (already received or planned to receive the vaccine). Racialized (self-identified as Asian, Black, Latinx, mixed-background) and Indigenous youth represented ~ 55% (*n* = 18) of participants who were not vaccine confident and only ~ 26% (*n* = 15) of those who were vaccine confident. Participants who believed they had a high chance of becoming infected with COVID-19 were more likely to be vaccine confident (*n* = 21; ~ 23%) versus non-confident (*n* = 6; ~ 7%).

### Odds ratios

Odds ratios are displayed in Table 5. Bivariate analyses were used to test the association between the outcome variable (vaccine confidence) and independent variables. In the bivariate analysis only racial-ethnic background and age were significant in relation to vaccine confidence. Table 5 illustrates differences in vaccine confidence among different groups. Although most being statistically non-significant these are exploratory to estimate vaccine confidence among these groups. Participants between 20 and 24 years and 25+ had 1.1 and 3.89 times the odds, respectively, of being vaccine non-confident in comparison to those under 19 years (OR = 1.1, 95% CI: 0.39-3.09; OR = 3.89, 95% CI: 1.2-12.64). Participants who reported their race as Black had 4.61 times the odds of being vaccine non-confident compared to non-Black participants (OR = 4.61, 95% CI: 1.42-15). Similarly, racialized participants had 4.73 times the odds of being vaccine non-confident compared to non-racialized participants (OR = 4.73, 95% CI: 1.88-11.39). Participants who scored mild/moderate or moderately severe/severe on the PHQ-9 depression scale had 2.74 times and 2.06 times the odds of being vaccine non-confident compared to those with minimal depression (OR = 2.74, 95% CI: 0.27-27.35; OR = 2.06, 95% CI = 0.21-19.79). Those with moderate/severe anxiety had 0.78 times the odds of being vaccine non-confident (OR = 0.78, 95% CI: 0.28-2.17). Participants who scored in the range of problematic substance use had 1.42 times the odds of being vaccine non-confident compared to those scoring in the non-problematic substance use range (OR = 1.42, 95% CI: 0.58-3.48).

**Table 5:** Odds Ratios for lack of confidence in the COVID-19 vaccine by sociodemographic and mental health characteristics

Age	OR	CI
15-19	reference	–
20-24	1.1	0.39-3.09
25+	3.89*	1.2-12.64
**Gender**		
Cisgender	reference	–
Transgender	0.46	0.15-1.43
Gender Diverse	1.08	0.39-2.98
**Employment Status**		
Paid employee and self-employed versus other categories	0.9	0.35-2.34
**Racial-Ethnic Background**		
White versus all other races	0.23**	0.09-0.56
Black versus all other races	4.61*	1.42-15
Asian versus all other races	0.54	0.14-2.17
Racialized versus non-racialized	4.73**	1.88-11.89
**Depression**		
Minimal	reference	–
Mild/Moderate	2.74	0.27-27.35
Moderately Severe/Severe	2.06	0.21-19.79
**Anxiety** (Ref Mild)		
Mild/Moderate	reference	–
Severe	0.78	0.28-2.17
**Substance Use**		
Non-problematic	reference	–
Problematic	1.42	0.58-3.48

*P < 0.05; **P < 0.001

The following themes emerged as the dominant factors associated with being vaccine non-confident among youth participants:

### Mistrust in the healthcare system

Mistrust in the healthcare system proved to be a dominant theme that was discussed among youth in relation to the COVID-19 vaccine. Youth who were vaccine non-confident were less likely to trust doctors, healthcare providers, public health agencies, and government websites. In addition, those who faced traumatic experiences engaging with the healthcare system reported broken trust, which often resulted in them avoiding medical institutions out of fear of being re-traumatized.

Participants described feeling dehumanized in their interactions with health providers and the healthcare system at large due to multiple intersections of their identity (e.g., homelessness, race, 2SLGBTQ+ identity). In the context of engaging with service providers, one participant stated *“Homeless people are almost never viewed as actual human beings. We are almost always viewed as subhuman.”* (Jamie, 21 years old). Due to this mistrust, youth discussed their fears of being experimented on with the vaccine: *“I didn’t want to be the first guinea pig.”* (Maira, 23 years old).

Racism was a prominent driver of mistrust in the context of both interpersonal experiences of discrimination within the healthcare system, as well as the medical system’s longstanding history of systemic mistreatment of racialized individuals. Participants expressed that this foundation of mistrust makes it difficult to work around uncertainties, such as those associated with the lack of long-term vaccine data and unclear public health messaging regarding vaccine rollouts. As one participant stated:

*“Honestly, I'm not an anti-vaxxer, I’m not an anti-masker. It's just, there is a history of the medical field and how they treat Black women and how they treat minorities in general, and I'm truthfully a little nervous about the vaccine that they give to disadvantaged people, just because what they've done in the past with vaccines and experimenting on Black women and that doesn’t seem super appealing to me. So, I do have worries about it...I just don't trust Western medicine. I don't, because it wasn't built for me. And it's not here to help me so I don't trust it.”* (Gabby, 17 years old)

### Lack of targeted vaccine-related information

Lack of vaccine-related information and mistrust in sources of information were key drivers of vaccine confidence. Almost half (42%, *n* = 14) of the participants who were vaccine non-confident reported that they do not trust the information they received about the COVID-19 vaccine; whereas, participants who were vaccine confident (100%, *n* = 58) all reported feeling neutral or trusting of COVID-19 vaccine information. Several youth described not following news about COVID-19. For example, one participant shared: *“My mom used to blare the news literally 24/7 at my house so it was hard to ignore. But now that I’m not at home I don’t check the news often.”* Summer (18 years old). For youth that did seek out information about COVID-19, the majority reported that they trust social media sources the most. For example, one participant stated *“I don’t listen to the news, 6ixBuzz [Canadian Media Platform] is my source of news.”* (Gabby, 17 years old). One key informant (emergency shelter) noted that youth might be receiving mixed messages when turning to social media for sources of news:

*“When you’re isolated and you don’t have a whole lot to do and all you have is electronics, all you’re seeing is media...there’s so much mixed messaging around what that looks like for them, and because there’s nothing concrete, they’re just saying no.”*

Following social media, youth also discussed word of mouth, and information shared within housing programs, as the most common sources of vaccine-related information. Numerous youth reported that they were not well informed about the vaccine rollout process (e.g., eligibility criteria, timelines). One participant shared:

*“The roll out process was a mess. I found all of, myself and my roommates, vaccine appointments via Twitter. The provincial government did a terrible job trying to explain how it all worked and whatnot. And I had to take on the task of booking appointments just because everyone else found it so difficult to try and navigate.”*

(Cassie, 26 years old)

When asked about public health messaging throughout the COVID-19 pandemic, the majority of interview participants reported that public health messaging was ineffective for their demographic and did not consider the needs and lived realities of youth experiencing homelessness. For example, one participant asked: *“How are you going to tell homeless people to stay home?”* (Ori, 24 years old). The gap in targeted messaging led another participant to state: *“People in their 20’s do not wear masks. They just don’t wear them. It needs to be advertised and publicised differently to target the market.”* (James, 25 years old).

One key informant (outreach) shared the importance of going beyond posting information about COVID-19 to having informed dialogue and conversations with young people:

*“I feel it should have been maybe doing literal outreach with nurses and firemen, paramedics… doctors, with community members doing outreach education… if you just give somebody a piece of paper with the information, they're not going to read that honestly. But if you give someone the paper, you tell them the information, and then they can ask you questions right then and there, and you make them feel safe, and you don'tmake them feel stupid for not for asking certain questions, then you'll probably be successful.”*

### Vaccine side effects

Participants who were vaccine non-confident reported more concerns about its unknown side effects, serious harmful effects, and long-term health consequences in comparison to participants who were vaccine confident. Youth were concerned about side effects for various reasons, including not wanting to be sick or being unable to fulfill daily responsibilities. Katniss, 22 years old, stated:

*“So now it's just been like trying to figure out how I would actually get it. And then if I got side effects and felt sick, how I would afford my dogs [being] taken care of during that time.”*

There also seemed to be a lack of clarity surrounding the legitimacy/safety of approved vaccines, which led to questioning and fear of side effects. One participant shared:

*“I’m actually in a clinical trial for a new one…. I was worried about getting Pfizer, because of all the symptoms that people could get, or Moderna, or all these other ones. So, it was kind of an easier way of me getting the vaccine, instead of having to get the ones that might not be qualified.”* (Riley, 21 years old)

### Accessibility

Accessibility concerns were also discussed as a barrier to receiving the COVID-19 vaccine. As one participant stated, geographical accessibility was an important underlying factor:

*“I would like to, but I don't know if I'll be able to, because I don't drive, and I can't get to places by myself. I have to rely on my mom to do that. And she has a very questionable theology of vaccines, so she probably won't want to do that unless she has to.”* (Jordan, 17 years old)

Participants also reported that vaccine-related information could have been made available in more accessible forms, including graphics, various languages, and catered to different learning styles, instead of utilizing a one-size-fits-all approach. As one participant shared:

*“In some high-risk areas, there's a linguistic and cultural barrier…. we need more public health messaging that is not English, like telling people to wash their hands regularly to physically distance, to mask, to sanitize... we need more culturally sensitive and culturally competent messaging.”* (CeCe, 26 years old)

Additionally, Edi, 20 years old, stated: *“I think more images or like, I don’t know, videos and stuff, maybe that would have helped better”.* Within the context of vaccine messaging, a key informant furthermore shared:

*“You’ve got to look at all the learning styles, barriers to understanding with instructions. The staff do a very good job of meeting people where they’re at and explaining where it is and what that means to them.”*

## Discussion

Our findings illustrate that the majority of youth participants felt safe receiving the COVID-19 vaccine and believed that the vaccine can curb the spread and protect them from COVID-19. However, we also found that numerous youth did not feel safe receiving the vaccine and were not planning to get vaccinated. Groups who lacked the most confidence in the vaccine included Black and racialized youth, which might be attributed to systemic racism, historical violence within healthcare, and institutional mistrust. As seen, this further exacerbated the race-based health inequities seen throughout the COVID-19 pandemic, where racialized individuals, particularly Black people, have been disproportionately affected by COVID-19 infections and deaths [29]. Other groups who lacked confidence in the COVID-19 vaccines included those aged > 25 years old; struggling with depression and/or substance use.

Our findings indicate that there are numerous factors associated with lack of confidence in the COVID-19 vaccine among 2SLGBTQ+ youth experiencing homelessness, including mistrust in the healthcare system, lack of targeted vaccine related information, uncertainty about side effects, and accessibility concerns. The World Health Organization (WHO) developed a framework called the three C’s (confidence, complacency and convenience) describing factors underlying and driving vaccine hesitancy [30]. This framework aligns closely with the themes found in our paper, and we will consider our themes in relation to this framework to gain a more robust understanding of vaccine confidence among 2SLGBTQ+ youth experiencing homelessness.

Informed by insights from key informants, who worked closely with vaccination programs, we identified several enablers to increasing vaccine uptake. The pervasive mistrust in the health system among this population necessitates the need to build and foster trust throughout the vaccine rollout process. As defined under “confidence” in the three C’s model, trust in the safety and efficacy of vaccines, the health system, and the motivations of policy makers are important drivers of vaccine confidence. With regards to information uptake, youth experiencing homelessness were more inclined to place confidence in information from trusted sources, which tended to exclude government sources and public health officials. Youth who have been unhoused or precariously housed often do not have a previous history of receiving vaccinations and/or being appropriately informed about them [31]. The majority of key informants agreed about the importance of engaging peer ambassadors to disseminate vaccine-related information, wherein, peers can share their personal experiences of getting vaccinated, and create safe spaces for dialogue to address any questions and concerns that youth may have. Within vaccination clinics, best practises include establishing a sense of familiarity by staffing the site with workers who have pre-existing trusting relationships with the youth and/or those who are trauma-informed and committed to understanding the lived realities and needs of youth facing various facets of marginalization. Involving primary care providers, who youth already have trusting relationships with, in vaccination rollouts and clinics may increase accessibility and vaccine confidence and uptake.

It is critical to address the accessibility needs of 2SLGBTQ+ youth experiencing homelessness in organizing vaccination rollouts, as highlighted under the “convenience” pillar of the WHO’s three C’s model. Convenience encompasses factors such as geographical accessibility, health literacy, and affordability. To address transportation barriers, several key informants echoed that vaccination clinics should be set up in easily accessible and consistent locations. Ensuring that youth have options to select vaccination location, time of day, and date, are key enablers of accessibility. Additionally, mental health challenges, such as depression, anxiety, suicidality, and substance use presents individual-level and structural barriers to vaccine uptake, impacting one’s perceptions about the severity of COVID-19, understanding of public health guidelines, and willingness to access preventative healthcare. Addressing mental health barriers is critical, given that 2SLGBTQ+ youth already experienced significant mental health disparities prior to the pandemic, and, it is known that people with severe mental health issues are at an increased risk of being infected with COVID-19 and experiencing poorer health outcomes (higher hospitalizations, morbidity, mortality) if infected [32]. Furthermore, a considerable number of youth did not perceive that COVID-19 was a risk to them, and this was cited as a reason for not prioritizing receiving the vaccine. Vaccination clinics that provided incentives, such as gift cards, were well received by youth, as this motivated them to get vaccinated in a timely manner.

Our findings suggest that the dissemination of vaccine and public health information could be improved for this demographic, as there was a lack of targeted messaging, poor consideration of the needs and lived realities of 2SLGBTQ+ youth experiencing homelessness, and lack of trust towards government and public health sources of information. This aligns under the three C model’s pillar “complacency”, wherein people perceive the risk of vaccine preventable diseases to be low and vaccination is not considered essential. Public health messaging about COVID-19 risks and vaccine rollout will be better received if delivered by service providers, community leaders, and healthcare practitioners who are trusted by 2SLGBTQ+ youth experiencing homelessness. Given that social media and word of mouth were the most prevalent sources of public health information for this demographic, targeted public health information could be further disseminated through these channels to better reach youth experiencing homelessness. Based on our interviews with youth and key informants, we also heard suggestions and recommendations to simplify public health information and provide more graphics to illustrate key concepts, consider and address various learning styles, provide translations in the languages commonly used in a given area, and create opportunities for two-way dialogue (forums, panel discussions).

This study is one of the first investigations exploring vaccine attitudes and uptake among 2SLGBTQ+ youth experiencing homelessness. Limitations include the cross-sectional nature of this study, and timing of data collection. Data was collected in a 6-month window between January and June 2021, a time during which there was significant change and development regarding COVID-19. During the early part of data collection (January-March 2021) vaccines were not readily available to the public, but became more accessible starting in April 2021. Due to the evolving epidemiology of COVID-19, the increase of public health messaging, and vaccine distribution efforts, predictors of vaccine confidence are likely to change overtime. Given the challenges recruiting this hard-to-reach population to participate in research, particularly during a pandemic, we argue that these insights have important practical significance. This study was limited to 2SLGBTQ+ youth experiencing homelessness in the Greater Toronto Area and surrounding areas, therefore, results may not be generalizable to the larger population of 2SLGBTQ+ youth experiencing homelessness, particularly those in rural settings.

## Conclusion

2SLGBTQ+ youth experiencing homelessness face multiple compounding layers of discrimination and disadvantage, and it is known that they have an increased risk of being infected with COVID-19 and will likely subsequently have a poorer prognosis and worse health outcomes due to their social determinants of health. Despite this serious concern, some 2SLGBTQ+ youth lacked confidence in the COVID-19 vaccine – primarily due to mistrust in the healthcare system, lack of targeted vaccine-related public health information, concerns about safety and side effects, and accessibility issues. It was also apparent that experiences of trauma and mental health challenges for this group resulted in some participants perceiving that COVID-19 was not as great of a personal risk to them, relative to their lived experiences, resulting in decreased vaccine priority. There is an ethical responsibility and urgent public health need to prioritize vaccinations among 2SLGBTQ+ youth experiencing homelessness, as this is one of many critical measures needed to address the health inequities facing this population, both during and after, the COVID-19 pandemic. When developing the COVID-19 vaccination strategy and rollouts, public health officials should consider prioritizing 2SLGBTQ+ youth experiencing homelessness through the following means: fostering trust through peer outreach programs to create safe spaces for dialogue and staffing vaccination clinics with trusted and familiar individuals; targeted public health messaging that centres the lived realities and needs of this demographic, and is delivered through channels most trusted and used by youth (i.e., community outreach, social media); and addressing accessibility needs, including setting up vaccination clinics in accessible locations and ensuring that various learning styles are addressed when delivering vaccine-related information.

## Supplementary Information

**Additional file 1.** Key Survey Measures.

## Data Availability

We are unable to make our data set publicly available for ethical reasons. This study involves sensitive human research participant data, which cannot be shared publicly. However, the Centre for Addiction and Mental Health (CAMH) Research Ethics Board can be contacted for future data request purposes: Dr. Robert Levitan, Chair, Research Ethics Board, Centre for Addiction and Mental Health, telephone: 416-535-8501 Ext 34020, email: robert.levitan@camh.ca

## Abbreviations

2SLGBTQ+: 2-spirit, lesbian, gay, bisexual, transgender, queer, and questioning
GTA: Greater Toronto Area
CAMH: Centre for Addiction and Mental Health CAB Community Advisory Board
CHERRIES: Checklist for Reporting Results of Internet E-Surveys
COREQ: Consolidated Criteria for Reporting Qualitative Studies
WHO: World Health Organization
