# Supplementary material for: Examining COVID-19 vaccine uptake and attitudes among 2SLGBTQ+ youth experiencing homelessness
Source: BMC Public Health. 2022 Jan 18;22:122. doi: 10.1186/s12889-022-12537-x (PMC8764500; doi:10.1186/s12889-022-12537-x)
Supplement: Supplementary file 1 — Additional file 1. Key Survey Measures. [file 12889_2022_12537_MOESM1_ESM.doc]

**Additional file 1: Key Survey Measures**

**COVID-19 Vaccine Questions**

1. Do you plan on receiving the COVID-19 vaccine?
   1. Yes
   2. No
   3. Unsure
   4. I have already received it
   5. Prefer not to answer
2. I trust that the information I receive about the COVID-19 vaccine is reliable and trustworthy
   1. Strongly Disagree
   2. Disagree
   3. Neutral
   4. Agree
   5. Strongly Agree
3. I worry about the unknown effects of the COVID-19 vaccine
   1. Strongly Disagree
   2. Disagree
   3. Neutral
   4. Agree
   5. Strongly Agree
4. I am concerned about serious harmful effects of vaccines
   1. Strongly Disagree
   2. Disagree
   3. Neutral
   4. Agree
   5. Strongly Agree
5. I am worried that the COVID-19 vaccine may lead to long-term health problems
   1. Strongly Disagree
   2. Disagree
   3. Neutral
   4. Agree
   5. Strongly Agree
6. I feel safe receiving the COVID-19 vaccine
   1. Strongly Disagree
   2. Disagree
   3. Neutral
   4. Agree
   5. Strongly Agree
7. I can rely on the COVID-19 vaccine to protect me from COVID-19
   1. Strongly Disagree
   2. Disagree
   3. Neutral
   4. Agree
   5. Strongly Agree
8. I believe the COVID-19 vaccine can curb the spread of COVID-19
   1. Strongly Disagree
   2. Disagree
   3. Neutral
   4. Agree
   5. Strongly Agree

**General Anxiety Disorder-7 (GAD-7)**

Over the last 2 weeks, how often have you been bothered by the following problems

1. Feeling nervous, anxious, or on edge
   1. Not at all
   2. Several days
   3. Over half the days
   4. Nearly every day
2. Not being able to stop or control worrying
   1. Not at all
   2. Several days
   3. Over half the days
   4. Nearly every day
3. Worrying too much about different things
   1. Not at all
   2. Several days
   3. Over half the days
   4. Nearly every day
4. Trouble relaxing
   1. Not at all
   2. Several days
   3. Over half the days
   4. Nearly every day
5. Being so restless that it's hard to sit still
   1. Not at all
   2. Several days
   3. Over half the days
   4. Nearly every day
6. Becoming easily annoyed or irritable
   1. Not at all
   2. Several days
   3. Over half the days
   4. Nearly every day
7. Feeling afraid as if something awful might happen
   1. Not at all
   2. Several days
   3. Over half the days
   4. Nearly every day

**Patient Health Questionnaire-9 (PHQ-9)**

Over the last 2 weeks, how often have you been bothered by any of the following problems?

1. Little interest or pleasure in doing things?
   1. Not al all
   2. Several days
   3. More than half the days
   4. Nearly every day
2. Feeling down, depressed, or hopeless?
   1. Not al all
   2. Several days
   3. More than half the days
   4. Nearly every day
3. Trouble falling or staying asleep, or sleeping too much?
   1. Not al all
   2. Several days
   3. More than half the days
   4. Nearly every day
4. Feeling tired or having little energy?
   1. Not al all
   2. Several days
   3. More than half the days
   4. Nearly every day
5. Poor appetite or overeating?
   1. Not al all
   2. Several days
   3. More than half the days
   4. Nearly every day
6. Feeling bad about yourself - or that you are a failure or have let yourself or your family down?
   1. Not al all
   2. Several days
   3. More than half the days
   4. Nearly every day
7. Trouble concentrating on things, such as reading the newspaper or watching television?
   1. Not al all
   2. Several days
   3. More than half the days
   4. Nearly every day
8. Moving or speaking so slowly that other people could have noticed? Or the opposite - being so fidgety or restless that you have been moving around a lot more than usual?
   1. Not al all
   2. Several days
   3. More than half the days
   4. Nearly every day
9. Thoughts that you would be better off dead, or of hurting yourself in some way?
10. Not al all
11. Several days
12. More than half the days
13. Nearly every day

**CAGE-AID**

1. Have you ever felt you ought to cut down on your drinking or drug use?
   1. Yes
   2. No
2. Have people annoyed you by criticizing your drinking or drug use?
   1. Yes
   2. No
3. Have you ever felt bad or guilty about your drinking or drug use?
   1. Yes
   2. No
4. Have you ever had a drink or used drugs first thing in the morning to steady your nerves or to get rid of a hangover?
   1. Yes
   2. No
